# The Annual Address Delivered to the Graduates of the Atlanta Medical College, at the Commencement, Sept. 2d., 1858

**Published:** 1858-10

**Authors:** Custis B. Nottingham

**Affiliations:** Macon, Ga.


ATLANTA
Medical and Surgical Journal.

Vol. IV.] OCTOBER, 1858. [No. II.

### ORIGINAL COMMUNICATIONS.

### ARTICLE I.

*The Annual Address delivered to the Graduates of the Atlanta Medical College, at the Commencement, September 2d,* 1858. By Custis B. Nottingham, M. D., of Macon, Ga. Published by the Faculty.

The importance of the Medical Profession, and the best means of attaining high position in it—subjects that have doubtless often engaged the thoughts of those gentlemen of my auditory, whom I have been invited specially to address—the Graduating Class of the present commencement—constitute a theme worthy of this interesting occasion.

The science of Medicine, looming up through the long vista of many centuries, from its chaotic and shadowy origin in the fabulous mythology of an era long anterior to that memorable event of antiquity—the Trojan war—baptised in the sacred rites of mystic Priestcraft—fashioned by the cold philosophy of Pythagorean teaching and prescription—strengthened and enriched by the anatomical investigations and acquirements of the Alexandrian School—remodeled and systematized by Grecian advancement and progress—cultivated and improved by Roman intelligence and wisdom—sustained and fostered by Arabian learning and conservatism, during that long gap in civilization, when Goth and Vandal violence overran all Europe, and

> "The world enrobed in midnight darkness slept,
> And gloomy vigils superstition kept;"

Coming forth from the cloister and monastery at the first dawn of literature, art and science, at the beginning of the fifteenth century, with as much of substantial merit as any of its associate sciences; elaborated, expanded, embellished and adorned by the genius and learning of the long line of profound thinkers, and active philanthropists, who have for the last five hundred years illustrated its annals, it stands forth in the middle of the nineteenth century, the living embodiment of the accumulated wisdom and sublime charities of more than three thousand years, commanding the confidence, the admiration and gratitude of the world—worthy indeed of all with which the mind in its superb majesty can invest it. Tracking the march of civilization and progress, intimately interwoven with the advancement of truth, knowledge and human happiness; partaking largely in its characteristics of the individuality and bitter philosophy of the various periods of its long and fluctuating history; hallowed in the self-sacrificing devotion of legions of the wise, the virtuous and the good, in every age and in all countries, its dignity and utility are now universally recognised and appreciated—wherever human cultivation obtains, commerce unfurls its flag, or Christianity plants its standard.

> "As some tall cliff that lifts its awful form,
> Swells from its vale, and midway leaves the storm,
> Tho 'round its breast the rolling clouds are spread
> Eternal sunshine settles on its head."

Measured by the exacting rule of practical value, applied in this utilitarian age to all science, art and institutions, that claim public attention, or engage private enterprise, the Medical Profession, as illustrated in the lives of its votaries, is of the first importance and highest dignity—useful to State and Empire in its benign influence on population; in diminishing the mortality of infantile life; in improving the health of the masses; and in extending the average term of life to increased years, it is of deep interest in a politico-economical point of view.

Sentineled on the outer walls of the social fabric, it is the trusted guardian of all classes and conditions of society—raising its warning voice against the violation of the well settled laws of life, devising and advocating the adoption and enforcement of sound and judicious hygienic and sanitary measures for the protection and preservation of the public health. Enshrined in the very bosom of domestic confidence, it is the ministering angel of high charities and God-like benefactions, alike in the palace of wealth and the hovel of poverty; in the sacred privacy of the family circle, and the public halls of philanthropy—every where calming fear, and allaying apprehension; relieving pain, and mitigating suffering; staying the hand of the destroyer, and rescuing from impending death valuable lives; lighting up the star of hope and cheerfulness, and beguiling the hours of sorrow and sadness—fortune and pauperism sharing in common the bliss of its rich stores of wisdom and expansive benevolence. Entering the retinue of an honorable, generous ambition, it braves the storms of ocean; contests the heart-chill of the icy atmosphere of the Poles; combats the fiery pestilence of the Tropics; ministers to the necessities of adventurer on land and sea; and consecrated in patriotic devotion, the wants of country, in defence or conquest, command its willing and priceless services for those of the chivalrous and daring of panoplied legions, who, amid the din, and smoke, and lurid glare, and carnage, and blood of battling hosts, fall for honor's sake.

Standing unmoved whilst others flee; when the bosom of desolation—the "pestilence that walketh in darkness, and the destruction that wasteth at noonday"—stalks boldly through densely populated cities; in those seasons of darkness and despair, when the cheeks of brave men turn pale; when the marts of business are forsaken; when the hammer of the artisan lies idle; the Courts of Justice are closed; the sounds of praise and prayer are no longer heard in the

Temples of the living God; when panic, and fever, and consternation, and alarm, prevail omnipotent; when men speak in whispers, and hushed silence holds universal sway —not only standing firm, but from early noon to dewy eve, and through the live-long night, with untiring patience, self-devotion, and moral heroism, and fearless daring—pursuing its quiet errands of mercy; seeking out the objects of its charities; ministering to the wants and sufferings of the afflicted; smoothing the pillow of the dying; consoling the anguish of the sorrowing, and interposing its strong arm as a break-water to turn back the tide of desolation and death; it challenges the homage of reason, and lays tribute upon the warmest affections of the heart—convincing by its triumphs, and charming by its celestial goodness.

The Ceremonies of this day, gentlemen of the graduating class of Atlanta Medical College, inaugurate a new era in your lives. Having been for many months, nay perhaps for long and wearisome years, engaged in laborious and noble efforts, aided by a corps of Instructors, whose learning and experience well qualify them for the responsible and arduous position which they occupy, to secure knowledge of the great truths and treasures of Medical science, you are now invested with the honors of the doctorate. Bidding adieu to the Halls of your Alma Mater, where you have been principally engaged in studying the theory of scientific medicine, you go forth to assume the responsibilities, encounter the perils, perform the duties, suffer the discomfitures, and reap the rewards of a participation in the active duties of practical life.

To the community, as I am assured by this large assemblage of civic worth and professional distinction, of age, youth, beauty and intelligence, the hour is one of no little interest. To you, undoubtedly, occupying for the first time this high stand-point in your lives, it is one of deepest concern —fraught as it is with the recollections of the past, the joys consummated of the present, and the hopes and anxieties of the future. Worthily successful in the attainment of the *chartered right* to embark upon the voyage which lies before you in a noble profession, all that lies outside the present ceremonials is as the land beyond the mountains, to Raselas in his peaceful and charming valley home. To some of you, I doubt not, the ocean upon which you are about to venture, seems rough and tempestuous—whitened with surging billows, foaming caps, and checkered with rocks and whirlpools, and quicksands, that often make shipwreck of the best appointed and most richly freighted barques; whilst to others, I presume, confident in untried powers, and elated with the illusions of youthful hopes, the sea seems smooth, the sky clear and serene, the sun bright and genial, the air stirred only by gentle zephyrs, and everything promising a safe, prosperous and happy voyage. As in nautical life, the returned mariner may impart information to those about to set out on voyages of discovery or commerce in seas to them unknown, that will improve their chart, and render their journey more safe and successful, so may I, perchance, having entered upon the same voyage years ago which you now contemplate, be able to offer you some suggestions—the result either of observation or experience—that will enable you the more safely and prosperously to pursue your future career.

Need I doubt that I address gentlemen who have a high, a noble purpose? Nay; the flashing eye, the beaming countenance, the well knit brow, the manly bearing, the high encomiums of your late teachers and oracles, but now peers and co-equals, satisfy me that in addressing the graduates of the present commencement, I speak to a body of well informed, intelligent, ambitious men, who will not be content with the empty title of Doctor, nor satisfied to hang as an incubus, or move in humble mediocrity on the outskirts of the profession of their choice; that I address gentlemen who are possessed of a proper sense of the dignity and usefulness of the time-honored pursuit in which they have resolved to engage, and are determined to

——— "Climb the hard ascent
Of high Parnassus—
Exulting o'er the painful steep to soar
High as the summit,"

And win for themselves a name and reputation that will, in coming years, command for them high position; fill the full measure of the hopes of kindred and friends; verify the flattering and kind predictions of these gentlemen, the faculty, who will watch their progress with almost paternal solicitude; and reflect back upon these halls with increased lustre much of the honors with which they have been this day clothed.

Aiming high, gentlemen, the achievement of success will require that your efforts shall be worthy of and commensurate with your aspirations. You must, through the powers and energy of mind and body, be the architects of whatever of fame and fortune you shall realize. Success will not result from accident, chance, or the favoring influence of external circumstances, but must be looked for in the manner in which you shape your conduct and character—in studious habits, industrious labor, energetic action; observation of the useful, self-control, honorable conduct, and generous benefactions—the highest reward of which is the rich and glowing consciousness of having done good. Your intellectual attainments and moral qualities will work out your destinies, and settle your permanent professional position uninfluenced to any considerable extent by the adventitious aids of fortune, family, friends, or social rank.

There is probably no error more common or more hurtful than that which results from the idea, that with the termination of the collegiate curriculum, and the awardment of a Diploma, ends the studious labors of those who aspire to professional distinction. It is true, the nature of education in Medical Schools differs from that common in more purely literary institutions, in the circumstance, that whilst in the latter, the chief object is the development, expansion, and discipline of the faculties of the mind; in the former, practical and useful knowledge; information having a direct bearing on that which is to be the every day business of life, is sought to be imparted; still, it is an opinion of universal recognition among those competent to judge, that at the conclusion of the short course of study, three years, required in American Medical Colleges, the foundation only of a good medical education is laid, and that those who would wish to be truly learned, well instructed in this, the most complex and difficult of the sciences, and among the most responsible of the pursuits known to civilized life, must add the superstructure, the body and finish of a thorough and complete education, by patient, careful and extensive reading; close, philosophical observation, and laborious discriminating reflection and deduction for many years after graduation. Not unfrequently is it the case, I apprehend, that some who have burnt the oil of the midnight lamp of their pupilage to good account, and enter the profession with fair attainments, and minds susceptible of the broadest cultivation—intellects competent to the highest efforts—genius quick, brilliant, and discursive, influenced by the pernicious and blighting idea so prevalent in the country, that their education was completed on issuing from the walls of their Alma Mater; or laboring under the equally suicidal delusion, that their native talent, with present acquirements, without subsequent study, will answer the purposes of high destiny, fritter away their lives in some humble position, when, with proper application, they could take rank among the first men—the brightest lights of their day and generation. If there be one of my auditors who, in looking forward to the consummation of this day's triumph, has cherished a thought so fatal to his ultimate good, he will allow me to warn him of its fallacy, and urge him to persevere in the habits of the student; else, when too late to repair the injury of irremediable negligence, "Memory will oft denounce the bitter curse of days misspent—talents misapplied, and fair occasions gone forever by." It was the memorable remark of the illustrious Rush, in retorting on a young physician, when full of the honors of a highly successful life, and his head whitened with the frosts of nearly seventy winters—"Sir, I am yet a student." Indeed, the domain of Medical Science is so broad and extensive—comprehending, as it does, a luminous philosophy in its several departments proper, besides levying tribute on nearly all the Natural Sciences, as auxiliaries in its literature—that even if complete and perfect as it now exists, it would afford an ample field for protracted study and elaborate inquiry; but when we note the fact, that it is not stationary, but steadily progressive—that its boundaries are being constantly enlarged; facts are contiually accumulating; errors are being corrected; and new and important truths are perpetually coming to light, it is the more apparent that to attain and preserve eminence, it is necessary that the physician should be a constant, a laborious student. Let not, then, the blighting mildew of indolence, the syren voice of pleasure, an interest in other business avocations, the labors and cares of even a large practice, nor the witching smiles or music of the bower, turn you from your purpose—your duty of study. But procuring in the outset of life a small and well selected library of standard authors; replenish it from time to time with valuable monographs, as they drop from the press; and subscribe for a few good periodical Journals; and for years to come, ever arranging your hours of business and pleasure with an eye to the improvement of your minds, and the acquisition of knowledge; master and treasure up from the best authors the rich and varied stores of existing information, evoke by your own thought from the great laboratory of nature some of her yet hidden mysteries; and peruse the monthly and quarterly publications with sufficient regularity to enable you to keep pace with the progress of the science. In your reading, let not the false glare of novelty, with its dazzling fallacies, captivate; neither let pre-conceived views, or blind obedience to the dicta magistre, obscure your mental vision to the impression of new light; but being cautious in adopting that which purports to be new, and still more cautious in rejecting that which has borne the test of long experience, and still commands the confidence of the well informed; contrast the views of one author with those of another; and these again with what you may have yourselves observed at the bedside. Thus, you will pursue the path of safety, intelligence, and certainty, and accumulate a fund of solid and valuable information, co-extensive and co-temporaneous with the scope and uttermost progress of your art, that will be a tower of strength—the Archimedean lever with which you will be able to prize up the valuable truths of science—the key with which you can unlock the great Arcana of disease. It will be the means of giving you promptness, decision, confidence and energy as practitioners, and commanding for you success in treating the numberless maladies in their many varying and protean forms, to which both mind and body are subject.

But, gentlemen, although intellectual culture is so important—yea, essential, to elevated position in the profession, this alone cannot acheive its attainment. The bright coruscations of genius and learning may for awhile captivate and dazzle as the meteors glare or lightnings blaze, but cannot command unreserved confidence, lasting success, or distinguished eminence recognized of all men. For the attainment of this, then, must be associated and blended with extensive and varied intellectual acquirements, the bright, steady, genial sunlight of pure and spotless morals, unstained by the vices, the immoralities, the dissipations so rife in the land. The medical man who expects to establish himself firmly in the affections of the public, and to secure high rank in a profession, distinguished in all ages for its bright exemplars of the more graceful virtues of the heart as well as for learning, must sustain a moral character, pure and without stain or blemish. Admitted into the inner sanctuary of domestic life; the only allowed observer behind the scenes that shut off the gaze of the world from the weakness and frailties of poor human nature when shorn of its mask of conventionality, diplomacy and disguise; the besought counsellor and only hope of man in his affliction, and woman in her peril; sharing, unreservedly, communications involving interests the most sacred, the tales of suffering and anguish, sorrow and woe, mental and physical, of all ages, sexes and conditions; the lone invited guest to the sacred apartment of innocence, beauty and loveliness, where the foot of sterner mortal, save that of father or husband, never trod before; enjoying indeed a confidence and toleration within his sphere of action possessed by no other member of community; the safety of society, the very instincts of nature, require that the physician should be a man eminently good and true—that he should be distinguished for uprightness of purpose, sincerity of heart, candor, prudence, truthfulness, integrity, a nice sense of high-toned honor, and a generous cultivation of the social graces, humane, kind and sympathizing in his intercourse with his patients; his character should be adorned in all its aspects, professional, moral and social, in the rich garniture of practical virtue, refinement and sublime philosophy, generous in his impulses, benevolent in his feelings, liberal in his sentiments; he should be chaste in his thoughts, pure and temperate in his habits, circumspect in his demeanor, discreet in conversation, wise in counsel, hopeful in temperament, cheerful in manners, self-sacrificing in devotion, true in his fidelity to confidence reposed, patient in giving attention to the long catalogue of sorrows, that may at times appear to him trifling, that overrun the hearts of those who narrate them, kind and philanthropic to the destitute, making a voluntary and free offering of much of his time and skill to the demands of charity, as presented in the persons of the sick poor wbo are to be found in every town and neighborhood. Perhaps there is no pursuit in which such unlimited and constant demands are made upon the benevolent feelings and charitable labors of its members, as are constantly pressed upon the medical profession. And whilst talent and industry should, in all the walks of life, command fair pecuniary remuneration, and whilst it may be even a question in moral ethics, whether any man has the abstract right, by his devotion to the claims of humanity or his unrequited contributions to the public, to make a pauper of himself in old age, or rear and turn loose on society a family of children unprovided for; still the true physician cannot pursue his high ministry under the demands of avarice—will not limit the exercise of his noble perogatives to the field of pecuniary return. The hope of amassing wealth, the desire of opulence, that great incentive and stimulus to thought and exertion with mankind, should never allure those whose sordid views do not extend beyond the gilded beauties of the almighty dollar to engage in this art, sharing the solemnities of the human destiny, and appendent, it may almost be said, to divinity itself, as an equal amount of talent and energy will pay much better in monetary emoluments in almost any other avocation; higher, nobler, more benevolent impulses should fire the ambition and energize the labors of the medical man. In all times our profession has been distinguished for its liberality, generosity, and philanthropy. The widow, the orphan, the decrepid, and the destitute, are yet the objects of its care and the recipients of its noble benefactions; and he, who, upon its threshold, has the range of his vision bounded by the horizon of mercenary considerations, and cannot view the field that opens before him, and invites the peril of comfort and health, and even life, with a wish to be a laborer therein for the sake of the pleasure and happiness it may afford him, for the sake of doing good, of making himself useful to his kind, and winning an honorable name, had best at once turn his attention to some other pursuit in which the labor will be more easy, and the substantial reward better. For such an one, the profession of medicine will be barren of delights, no pleasures are in view, no honors beckon onward, no rewards are in store, no trophies await. But to him in whose bosom burns the spirit of an enlarged philanthropy, who aspires to the high distinction of finding his greatest happiness in generous benefactions, who is desirous of emulating the illustrious hosts of the past and the present, in making a votive offering of himself on the altar of humanity, and who can be content with a pecuniary competency; to him the field is inviting, and if, with untireing zeal and unabating labor, he persues his noble calling, under the guidance of intelligence and high moral principle, he cannot fail to acheive the proud consciousness of a life well spent, nor be disappointed in securing the regard and respect of the community, winning the love and gratitude of a large circle of attached friends and obtaining a sufficiency of the more substantial rewards of labor, to serve the requirements of a moderate independence.

Self control, gentlemen, is indispensable to high position or eminent success. Man is a marvelous combination of earth and spirit, a strange, philosophically, incomprehensible "compound of dust and deity," and the weakness and frailties of the body are ever at war with the interests and advancement of the mind. The love of self indulgent ease, the fondness of pleasure, the demands of selfishness, the disposition to defer and procrastinate in the time of duty, inability to restrain the appetites, and alluring temptations to the positive vices beset the path and fetter the spirit in every honorable pursuit. The power to shake off these clogs to progress, to bring the feelings into subjection to the judgment, to enthrone proud reason and give it will and sway, is a necessity to him who would win honor and fame in the higher walks of the profession. The life of the physician being one of constant labor, toil and care, the demands upon his attention, if regularly, promptly, and continually responded to, as they should and must be, if he expects to carve for himself a name above mediocrity, will leave but little time to be appropriated to the gaieties of pleasure, to be whiled away in indolence, or to be swallowed up in revelry and dissipation. To warn you, gentlemen, against the enemy that lurketh in the wine cup, would, I trust, be a work of supererogation; it would probably be an impeachment of your intelligence, and a misgiving of your fealty to the wise and safe counsels of your earlier years; for who so dull, who so callous, who so heartless as to look upon the bleared eye, the be-sotted face, the wanton lip, the tottering step, the be-clouded intellect, or listen to the ribald jest or maudling talk of the debauched physician, without mortification and sorrow, loathing and contempt, fear and trembling; better that such an one had never been born, or had found a grave in youth or manhood's early prime, than to take upon himself the grave responsibilities of medical arbiter in the issues of life and death.

Candor and conscientiousness, are cardinal virtues in all the relations of life; in none are they more so than in that subsisting between physician and patient. A sacred regard for truth and honesty should ever mark the conduct of the medical practitioner. If the acknowledgement of error be manly, a conscientious deference to truth and justice and a frank avowal that may prevent the commission of error cannot be otherwise than honorable in itself and must ultimately if not immediately, result in the bestowal of merited reward.

In your future, gentlemen, it may doubtless be your fortune to meet with cases of disease difficult of diagnosis, some, indeed, involved in so much obscurity as utterly to defy solution. Hurried and false diagnosis, with the mischievous and sometimes disastrous treatment instituted thereon, would not only be unjust to the patient, but hurtful to the reputation and prospects of the physician. To the investigation of such cases, if desirous of winning and maintaining reputation, bring intelligent, patient, laborious inquiry, aided by the lights of scientific reading, if the emergency will admit of it, and unravel their intricacies and lay bare their true pathological state before announcing an opinion or venturing to invoke the agency of therapeutics, and should you finally, as will sometimes probably occur, be unable to master the task of rational and satisfactory diagnosis, rather than subject the sufferer to hurtful and improper treatment, and yourselves to the mortification—perchance remorse—of having unwisely tampered with the great interests of life, at once frankly state your embarrassment to invalid or friends and request the aid of counsel or allow them the opportunity of seeking advice elsewhere; however humiliating to the genius of medical science, however mortifying to our own pride it may be, it is, nevertheless, sadly true, and the fact stares us constantly in the face, that there are yet forms of disease, the opprobia of the profession, for the cure of which our art is powerless, impotent. For the mitigation of suffering we may do much—for their cure we should be very sparing of promises. They constitute a fertile field for the exercise of empiricism and charlatanry, afford a soil in which the *isms* and *pathies* and *secret nostrum, patentee-ism* of the day most luxuriantly flourish, but those who substitute cunning and duplicity for sincerity, or claim the possession of some secret Panacea in dealing with the unhappy victims of such maladies, may appease their craving love of mammon and fill their purses with ill-gotten gains, but can never occupy the high ground of professional distinction, and after a short lived career of low craft and perfidy will pass from view, with the curses of disappointed hopes resting on their heads, the association of the vile encompassing their paths, the finger of contempt and scorn pointing to their memory, and a legacy of dishonor and bad fortune cleaving to their descendents. To a true physician a character of honor and integrity is all that "virtue is to a woman, or courage to the soldier;" having unusual, almost unlimited, privileges in looking in upon the domestic concerns and arrangements of families; the constant observer in the sacred precincts of the sick chamber of mother, wife and daughter in hours of grief, suffering and peril; the lone confident of unbosomed secrets, in the narration of which manhood trembles, beauty blushes, and innocence stands appalled; his reputation for prudence, circumspection and discretion should be a guarantee of safety; he should, in a word, be the pink of honor, and the personification of fidelity. To abuse confidence thus reposed, to babble of matters thus observed, to make commerce of matters thus obtained' to betray secrets thus possessed, cannot otherwise than involve the loss of the respect and confidence of the intelligent, refined and virtuous of community. So sacred do the good and pure of the profession consider revelations thus made, that many, and among them are some of the brightest ornaments of human kind—sustained by distinguished jurists—deem even the solemn exactions of an oath, in a court of justice, insufficient excuse for its disclosure or betrayal. And although the recognition of a "higher moral law" of binding force, might, in the judgment of the speaker, be subversive of the ends of justice and detrimental to the public weal, still, all concur in the conviction that nothing short of the solemnity and sanctity of such a tribunal should tempt the upright physician to unveil the many delicate and private matters that come under his view and reach his ear in the discharge of his professional duty.

In conclusion, gentlemen, allow me to suggest, that whilst servile imitation of manners, a silly copying of eccentricity of habit or a blind obedience of judgment to the authority of others should be avoided; that whilst every man should form and entertain and be prepared to express and defend his well matured opinions, and not allow others to do his thinking for him; yet as a means of success, manly, honorable and ennobling, that you select and have constantly in view, for *emulation*, some high standard of merit as illustrated and personified in the lives of some of the gifted, the learned, the wise and the good, who have, with the march of time, in all ages, adorned and shed lustre on the profession. Let this standard be a model one in all the great qualities of the true and upright physician, let it be of the fairest proportions, the most perfect symmetry and completest finish, the very *beau ideal* of excellence and greatness, not of such as have loitered about the portals or in the vestibule, but of those who have worshiped and ministered at the altars of the inner courts of the temple of your science.

Having selected your model, reject all compromises with indolence and ease, eschew the pretentions of the uncandid and the cunning, contemn all that is disingenuous and unmanly, abjure all dalliance with the various "Will of the Wisp" lights in the form of new systems that ever and anon rise up to shed their temporary treacherous glare on the tried pathway of legitimate medicine, and offer some short but delusive road to success, such as the royal pupil of Euclid would have been pleased to tread in science, and girding on the polished armor of your present acquirements, burnishing the shield of your lofty resolutions, and brightening the scimitar of your devotion and zeal, enter the list with the fixed purpose of conquest, and as the Bird of Jove in storm or sunshine fixes his piercing eye on his eyry high up in some mountain cliff, with untiring pinion wings his way upward and onward, and still upward until he gains his skyward home above the clouds, so may you attain an eminence far above the less aspiring, from which, in advanced years, you may calmly survey the struggles—the labors of the great battle fought, the victory won, and enjoy the jubilant acclaim of "benefactors of your species, beacon-lights in the pathway of humanity and heralds of an age of progress," whose honest fame will be destined to live beyond the grave. But if in emulating so eminent and exalted a model any of you should not be able to climb past the legions of minds of mark that are busily engaged in toiling on every high-road to pre-eminence in these times of universal intellectual struggle and triumph, if you should fail to realize to its full extent the bright and golden dreams of your ambition, if your hopes of equalizing or surpassing one admired intellectual and moral Colassus, of world-wide renown should not be consumated in full fruition; still aiming high and carrying into the heated contest the capabilities and acquirements, the industry and perseverance, the virtues and graces, which It has been my object briefly to delineate, you cannot fall short of achieving a degree of success that will command for you prominence among your peers, obtain the respect and the regard of the public whom you may serve, win the affections of numerous friends, secure the calm serenity and happiness of a well spent life, and when, after years of usefulness and philanthropy, you shall ultimately pay the great debt of nature, a summons from which there is no appeal, and which must soon or late come to us all, your good deeds will still live—your names will be consecrated in family benedictions—your memory will be hallowed in the heart's benisons of the poor, and cherished in the grateful recollections of the intelligent and good; over your bier the hand of beauty will spread its garlands of roses and laurel, and bereaved manhood keep its sorrowing vigils; tears of gratitude will water the bay that may bloom at your head and the willow that shall weep at your feet, and the smiles of an approving Heaven shall illumine the sanctity of your tomb.

Then—

"Press on, there's no such word as fail;
Press nobly on, the goal is near;
Ascend the mountain; breast the gale;
Look upward, onward, never fear."

